# Context Modulates the Contribution of Time and Space in Causal Inference

**DOI:** 10.3389/fpsyg.2012.00371

**Published:** 2012-10-01

**Authors:** Adam J. Woods, Matthew Lehet, Anjan Chatterjee

**Affiliations:** ^1^Center for Cognitive Neuroscience, University of PennsylvaniaPhiladelphia, PA, USA; ^2^Department of Neurology, Center for Functional Neuroimaging, University of PennsylvaniaPhiladelphia, PA, USA

**Keywords:** causality, causal inference and perception, contextual information, decision-making, time, space, temporal contiguity, spatial continuity

## Abstract

Humans use kinematic temporal and spatial information from the environment to infer the causal dynamics (e.g., force) of an event. We hypothesize that the basis for these inferences are malleable and modulated by contextual temporal and spatial information. Specifically, the present research investigates whether the extent of a person’s ongoing experience with direct causal events (e.g., temporally contiguous and spatially continuous) alters their use of time and space in judgments of causality. Participants made inferences of causality on animated launching events depicting a blue ball colliding with and then “launching” a red ball. We parametrically manipulated temporal contiguity and spatial continuity by varying the duration of contact between the balls and the angle of the second ball’s movement. We manipulated participants’ level of exposure to direct causal events (i.e., events with no delay or angle change) between experiments (Experiment 1: 2%, Experiment 2: 25%, Experiment 3: 75%). We found that participants adjust the temporal and spatial parameters they use to judge causality to accommodate the context in which they apprehended launching events. Participants became more conservative in their use of temporal and spatial parameters to judge causality as their exposure to direct causal events increased. People use time and space flexibly to infer causality based on their ongoing experiences. Such flexibility in making causal inferences may have adaptive significance.

## Introduction

The ability to infer causal structure in events is a central feature of human cognition (e.g., Hume, 1740/1960,1748/1977; Michotte, 1946/1963). Many researchers argue that the ability to infer causal relationships in physical and social events is an innate facet of human cognitive systems (e.g., Michotte, 1946/1963; Leslie, 1982, 1984; Leslie and Keeble, 1987; Oakes and Cohen, 1990; Scholl and Tremoulet, 2000; Blakemore et al., 2001; Wolpert, 2003, 2006, 2009). This ability allows us to understand relationships in our environment, predict future outcomes, and plan goal-directed actions. Wolpert (2003, 2009 argues that causal inferences set humans apart from animals and was critical in the evolution of *Homo sapiens*.

We use kinematic information, like time and space, to infer the dynamic properties of an event. In other words, we use visible parameters to make inferences about invisible forces (i.e., dynamics). Wolff (2007, 2008) suggests that we make causality judgments based on such inferences of invisible forces. The notion of force is of course derived from the apprehension of acceleration (*f* = ma; *f* = force, *m* = mass) that itself is dependent on how an object changes in time and space (*a* = Δv/Δt; *v* = Δd/Δt; *a* = acceleration, *v* = velocity, *d* = displacement in space, *t* = interval of time). The kinematic properties of objects in time and space fundamentally contribute to our judgments of causality in mechanical events (Schlottmann and Shanks, 1992; Scholl and Tremoulet, 2000; Blakemore et al., 2003; Guski and Troje, 2003; Roser et al., 2005; Schlottmann et al., 2006; Wolff, 2007, 2008; Buehner and Humphreys, 2010).

The present research investigates the mapping of time and space on to causal judgments. Specifically, we examine the role of the context in which participants apprehend kinematic temporal and spatial information. Contextual information strongly modulates human decision-making (e.g., Rohrbaugh and Shanteau, 1999; De Martino et al., 2006; Dror et al., 2006) by allowing us to integrate relevant proximate information. It plays an important role in how we interpret events and plan appropriate responses. For example, imagine a person standing in a room with a dangerous animal. What would you infer to be the person’s next action? What if the person standing in the room is a zookeeper or the animal is inside a cage? Contextual information alters our interpretation of the relationship between objects in an event, as well as our predictions of the actions and subsequent reactions of the objects. The same appears to be true for inferences of causality in events (e.g., Gruber et al., 1957; Powesland, 1959; Shanks, 1985; Schlottmann, 1999; Buehner and May, 2002, 2003).

Previous research suggests that contextual information provided by foreknowledge about the temporal characteristics of an event can influence how we interpret the relationship between time and causality (e.g., Schlottmann, 1999; Buehner and May, 2002). Schlottmann (1999) and Buehner and May (2002, 2003) demonstrated that the role of temporal information in causal inferences is mediated by people’s assumptions about the timeframe of events. For example, when people expect a delay in events, they expand the temporal delays they are willing to incorporate in their causal inferences. That is to say, people are willing to bridge the temporal gap in the event and infer a causal relationship.

Gruber et al. (1957) and Powesland (1959) also found that prior experience with clearly causal or non-causal events alters people’s representation of the relationship between time and causality. Gruber et al. (1957) found that providing participants with prior experience on practice trials demonstrating large violations of temporal contiguity (i.e., time delays) in a bridge collapse event relaxed subsequent temporal criteria for causal judgments. Powesland (1959) found that previous experience with practice trials demonstrating causal events without violations of kinematic temporal information (i.e., no time delay) made subsequent temporal criteria for causal judgments more conservative. Furthermore, Powesland demonstrated that inserting a series of example trials between blocks of events also influenced temporal criteria used to make causal judgments. Collectively, these data suggest that contextual information influences our interpretation of the relationship between time and causality.

While the use of temporal information to infer causality appears susceptible to context, the susceptibility of spatial information to context remains unknown. Furthermore, although prior experience and foreknowledge influence inferences of causality, it remains unclear whether ongoing exposure to contextual information modulates the contributions of time and space to causal inferences. In the present study, we focus on the role of contextual information in causal inferences using depictions of simple mechanical events (e.g., two balls colliding). We hypothesize that contextual information modulates the use of kinematic temporal *and* spatial information when inferring causality. The present study focuses on two aspects of temporal contiguity and spatial continuity: temporal delay and linearity of movement. In the present study, we specifically investigate whether contextual information provided by recent and ongoing experience with direct causal events (a mechanical event depicting a linear collision without any delay) influences participants’ judgments of how time and space contributes to causality. Consistent with Powesland’s (1959) findings, we propose that the proportion of recent experience with direct causal events will lead participants to interpret the relationship between time, *space*, and causality more conservatively. That is to say, people with increased exposure to direct causal events will only accept smaller violations of time and space as causal. Such a finding would suggest malleability of the use of time and space in judgments of causality, specifically in response to ongoing changing dynamics in a sequence of events.

To test our hypotheses, we varied the proportion of direct causal events in three experiments (Experiment 1: 2%, Experiment 2: 25%, Experiment 3: 75%). Unlike previous research, we manipulated the probability of exposure to direct causal events during the actual experiment, rather than with previous experience (e.g., practice trials; Powesland, 1959) or foreknowledge of underlying mechanisms (e.g., Schlottmann, 1999; Buehner and May, 2002, 2003). Thus, changes in the contribution of kinematic temporal and spatial information to causal inferences would reflect response to recent and ongoing experience with clearly causal events. Furthermore, the present research extends our understanding of the role of spatial information in causality, whereas previous research only investigated the influence of temporal contextual information. Experiment 1 provided a baseline for comparison of the context manipulation and evaluated participants’ general representation of the relationship between time, space, and causality. Experiment 2 investigated whether an increase in proportion of exposure to direct causal events modulates the contribution of time and space to causal inferences (i.e., causal context). Finally, Experiment 3 sought to extend findings from Experiment 2 by exposing participants to trials predominantly composed of direct causal events.

## Experiment 1

We presented participants with *launching events* containing parametric manipulations of time and space relevant to causality. Launching events have a long history in the study of causality (e.g., Hume, 1740/1960,1748/1977; Michotte, 1946/1963; Scholl and Tremoulet, 2000, etc.). These simple mechanical events portray one ball moving toward, contacting, and *launching* a second ball into motion. An increase in the time between initial contact of the first object and the initial movement of the second object or an increase in the deviation of the angle of egress for the second object decreases the likelihood of causal perception (e.g., see for examples Schlottmann and Anderson, 1993; Straube and Chatterjee, 2010).

Participants in Experiment 1 made inferences of causality on 98 launching events. Only two of the 98 launching events depicted direct causal launches (no time delay, no change in angle). The remaining 96 trials depicted a combination of parametric variations in temporal and spatial continuity.

### Materials and methods

#### Participants

Sixteen right-handed, native English speaking college students at the University of Pennsylvania participated in Experiment 1. All participants had normal or corrected to normal vision and were naïve to the purposes of the experiment. All participants gave written informed consent prior to participation in the study. The University of Pennsylvania’s Institutional Review Board approved the study.

#### Materials

**Figure 1:**
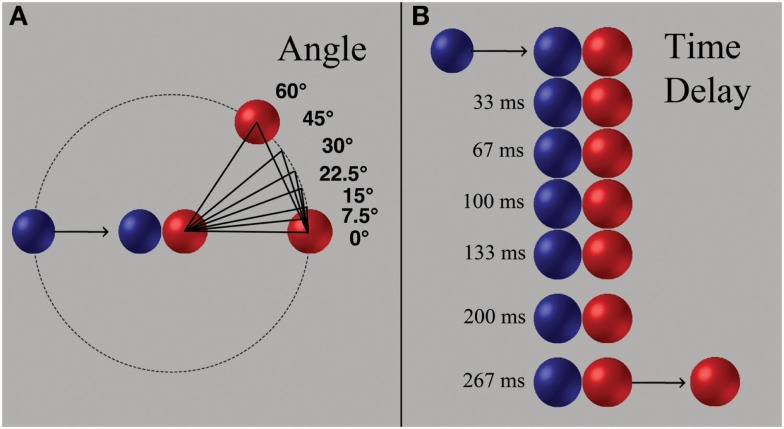
**(A)** Parametric manipulations of spatial continuity and **(B)** temporal contiguity common to all experiments. Balls on the left were blue (*r* = 14, *g* = 5, *b* = 223), balls on the right were red (*r* = 255, *g* = 0, *b* = 0), and the background was gray (*r* = 192, *g* = 192, *b* = 192).

Stimuli were 2 s animated video clips, generated in Strata 3D, depicting a blue ball colliding with a red ball (i.e., a launching event). Contact of the blue ball then “launched” the red ball (Figure 1). Temporal contiguity was parametrically varied between the contact of the blue ball and initial movement of the red ball (seven time delays: 0, 33, 67, 100, 133, 200, 267 ms). Spatial continuity was parametrically varied by changing the angle of egress of the red ball (seven angles: 0°, 7.5°, 15°, 22.5°, 30°, 45°, and 60°). The speed (9 cm/s), distance traveled (4.5 cm), and size (1.5 cm diameter) of each ball were constant. Videos were presented using Presentation experimentation software on a Windows XP computer.

#### Experimental design

Stimuli from Experiment 1 presented launching events where the blue ball approached the red ball along the horizontal axis (see Figure 1A). Upon contact of the blue ball, the time delay of initial movement of the red ball varied (Figure 1B) followed by variation in its angle of egress (Figure 1A). All possible combinations of time delays and angle changes resulted in 49 different stimulus conditions. Each stimulus was presented twice (total trials = 98). Only two of the 98 events demonstrated direct causal events (i.e., no time delay or change in angle). Videos were presented in random order. Each video was followed by a fixation cross with a variable duration of 2–8 s (average 5 s). Testing time in Experiment 1 was ∼12 min.

#### Procedures

Participants judged the causal relationship between the balls using a two-alternative forced choice design (Instructions: In every video, you will see a blue object and a red object move across the screen. You will be asked to judge whether the blue object caused the red object to move. We are interested only in your perception. There are no right or wrong answers. Please respond as quickly as possible to each video. Press “index finger” if you believe the blue object caused the red object to move. Press “middle finger” if you do not). Participants responded with the dominant hand and were asked to push a button with the index finger (Yes/causal) or middle finger (No/non-causal). Participants were first exposed to six representative practice trials before proceeding with the test trials. Practice trials were the same for all three experiments in the present manuscript and only one of the six trials demonstrated a direct causal launch. All participants were tested in a quiet testing room with the door closed to prevent distraction.

#### Analyses

In all experiments, generalized linear mixed models (GLMM) were applied to evaluate the contribution of changes in time delay and in angle to the odds of making a causal judgment, which explicitly model within-subject correlation by using subject-specific random effects. GLMM were calculated using the Proc NLMIXED procedure in SAS and variables were non-centered. Time delay and angle changes were coded in milliseconds and degrees, respectively. Trial number was included in GLMM to evaluate effects of experience over time. This factor is particularly important because each of the three present experiments contain different numbers of total trials. Parameter estimates from GLMM were evaluated for both sign and significance as an indication of sensitivity to variation in violations of time and space and significant use of either time or space for the judgment of causality, respectively. Significant Trial × Time or Trial × Space interactions would suggest that experience with launching events over the duration of the experiment influenced participants’ inferences of causality.

### Results

**Figure 2:**
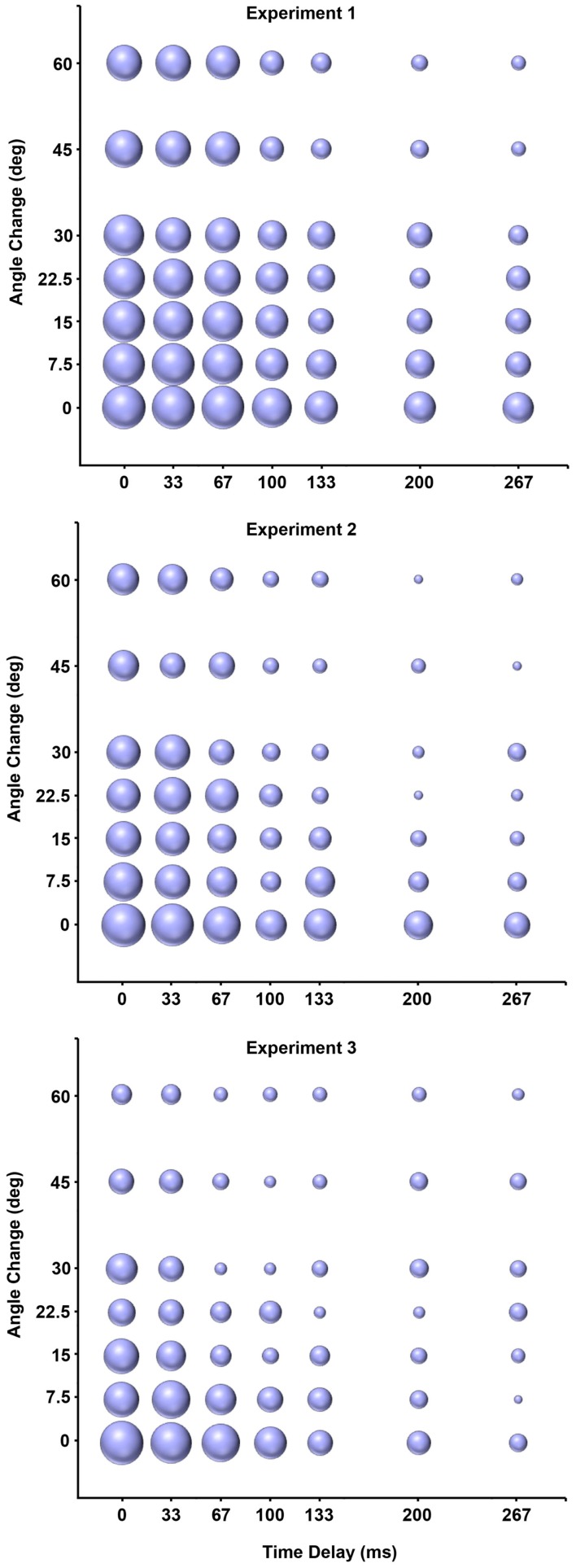
**Change in the percent of temporal and spatial parameter combinations judged causal across experiments (Top: Experiment 1, Middle: Experiment 2; Bottom: Experiment 3)**. Bubble area represents the percentage of a unique parameter combination (e.g., 33 ms delay × 15° angle change) judged causal. Increased bubble sizes for shorter delays and smaller angle changes, relative to longer delays, and angle changes, demonstrates that participants were more likely to judge shorter delays and angles as causal. Decreased size of bubbles across experiments demonstrates the effects of increased exposure to direct causal launches across experiments. Means for direct launches were calculated based on different numbers of trials in each experiment (Experiment 1 = 2; Experiment 2 = 32; Experiment 3 = 288 trials).

Generalized linear mixed models analysis demonstrated that participants used kinematic temporal and spatial information to infer causality (see Table 1 for parameter estimates). There was no main effect of Trial, nor any significant interactions (e.g., Trial × Time, Trial × Space, Time × Space; Table 1). Negative parameter estimates in main effects of Time and Space demonstrate that participants were more likely to judge an event as causal with smaller time delays and angle deviations (see top panel of Figure 2; see Table 2 for mean values).

**Table 1:** **Parameter estimates for generalized linear mixed models**.

	Experiment 1 (DF = 15)			Experiment 2 (DF = 15)			Experiment 1 vs. 2 (DF = 31)			Experiment 3 (DF = 15)			All experiments (DF = 47)		
	Est. ± SE	*t*	*p*	Est. ± SE	*t*	*p*	Est. ± SE	*t*	*p*	Est. ± SE	*t*	*p*	Est. ± SE	*t*	*p*
Intercept	2.0 ± 0.42	4.8	<0.001*	2.4 ± 0.34	7.3	<0.001*	0.66 ± 0.91	0.72	0.475	2.6 ± 0.21	12.6	<0.001*	2.1 ± 0.56	3.7	<0.001*
Context	−	−	−	−	−	−	0.98 ± 0.53	1.8	0.078	−	−	−	0.17 ± 0.22	0.78	0.441
Trial	0.002 ± 0.006	0.36	0.725	−0.002 ± 0.003	−0.72	0.480	0.01 ± 0.01	1.3	0.195	0.002 ± 6E−4	4.7	<0.001*	−0.005 ± 0.007	−0.79	0.433
Time	−0.009 ± 0.002	−3.8	0.001*	−0.01 ± 0.002	−6.4	<0.001*	−3E−4 ± 0.005	−0.06	0.955	−0.02 ± 0.002	−9.0	<0.001*	−0.006 ± 0.003	−1.6	0.100
Space	−0.02 ± 0.01	−2.3	0.036*	−0.05 ± 0.009	−5.5	<0.001*	0.02 ± 0.02	1.0	0.327	−0.09 ± 0.008	−10.1	<0.001*	−0.002 ± 0.01	−0.14	0.885
Context × trial	−	−	−	−	−	−	−0.01 ± 0.007	−1.4	0.156	−	−	−	0.002 ± 0.002	1.1	0.250
Context × time	−	−	−	−	−	−	−0.008 ± 0.003	−2.2	0.032*	−	−	−	−0.004 ± 0.002	−2.8	0.006*
Context × space	−	−	−	−	−	−	−0.03 ± 0.01	−2.7	0.011*	−	−	−	−0.02 ± 0.006	−4.1	<0.001*
Trial × time	−3E−5 ± 4E−5	−0.64	0.533	−5E−5 ± 3E−5	−1.5	0.142	−7E−5 ± 9E−5	−0.72	0.477	−5E−6 ± 1E−5	−5.2	<0.001*	−1E−6 ± 6E−5	−0.02	0.981
Trial × space	−9E−5 ± 1E−4	−0.45	0.656	−5E−5 ± 1E−4	−0.42	0.681	−4E−4 ± 4E−4	−0.94	0.352	−1E−4 ± 4E−5	−3.4	0.003*	1E−4 ± 2E−4	0.73	0.469
Time × space	−4E−5 ± 6E−5	−0.45	0.656	−9E−5 ± 1E−4	−0.92	0.371	−8E−5 ± 2E−4	−0.44	0.667	3E−4 ± 7E−5	4.3	<0.001*	−2E−4 ± 1E−4	−1.4	0.168
Context × trial × time	−	−	−	−	−	−	1E−5 ± 5E−5	0.23	0.821	−	−	−	−2E−5 ± 2E−5	−0.81	0.421
Context × trial × space	−	−	−	−	−	−	2E−4 ± 2E−4	0.78	0.441	−	−	−	−1E−4 ± 8E−5	−1.4	0.159
Context × time × space	−	−	−	−	−	−	4E−6 ± 1E−4	0.03	0.976	−	−	−	1E−4 ± 6E−5	2.4	0.017*
Trial × time × space	−3E−7 ± 1E−6	−0.21	0.839	2E−6 ± 1E−6	1.9	0.069	−2E−6 ± 3E−6	−0.51	0.612	1E−6 ± 4E−7	4.1	<0.001*	−2E−6 ± 2E−6	−0.68	0.500
Context × trial × time × space	−	−	−	−	−	−	2E−6 ± 2E−6	1.0	0.311	−	−	−	1E−6 ± 8E−7	1.4	0.1433

*Significance at *p* < 0.05; Est., parameter estimate; SE, standard error.

**Table 2:** **Percent of parameter combinations judged causal by experiment**.

Angle (°)	Delay (ms)	Experiment 1	Experiment 2	Experiment 3	Angle (°)	Delay (ms)	Experiment 1	Experiment 2	Experiment 3
		Percent ± SE	Percent ± SE	Percent ± SE			Percent ± SE	Percent ± SE	Percent ± SE
0	0	94 ± 4	96 ± 1	96 ± 0.3	22.5	133	38 ± 9	13 ± 6	6 ± 4
0	33	91 ± 5	91 ± 5	84 ± 7	22.5	200	19 ± 7	3 ± 3	6 ± 4
0	67	91 ± 5	71 ± 8	72 ± 8	22.5	267	28 ± 8	6 ± 4	16 ± 7
0	100	78 ± 7	47 ± 9	52 ± 9	30	0	81 ± 7	56 ± 9	48 ± 9
0	133	53 ± 9	52 ± 9	31 ± 8	30	33	63 ± 9	63 ± 9	31 ± 8
0	200	50 ± 9	41 ± 9	28 ± 8	30	67	59 ± 9	31 ± 8	6 ± 4
0	267	47 ± 9	32 ± 9	16 ± 7	30	100	41 ± 9	16 ± 7	6 ± 4
7.5	0	88 ± 6	75 ± 8	63 ± 9	30	133	38 ± 9	13 ± 6	13 ± 6
7.5	33	91 ± 5	61 ± 9	75 ± 8	30	200	31 ± 8	6 ± 4	17 ± 7
7.5	67	81 ± 7	47 ± 9	47 ± 9	30	267	19 ± 7	16 ± 7	13 ± 6
7.5	100	53 ± 9	19 ± 7	32 ± 9	45	0	69 ± 8	47 ± 9	31 ± 8
7.5	133	44 ± 9	44 ± 9	28 ± 8	45	33	63 ± 9	31 ± 8	28 ± 8
7.5	200	41 ± 9	19 ± 7	16 ± 7	45	67	59 ± 9	34 ± 9	13 ± 6
7.5	267	31 ± 8	16 ± 7	3 ± 3	45	100	28 ± 8	13 ± 6	6 ± 4
15	0	88 ± 6	61 ± 9	63 ± 9	45	133	19 ± 7	10 ± 5	9 ± 5
15	33	78 ± 7	56 ± 9	44 ± 9	45	200	16 ± 7	9 ± 5	16 ± 7
15	67	81 ± 7	41 ± 9	22 ± 7	45	267	9 ± 5	3 ± 3	13 ± 6
15	100	53 ± 9	22 ± 7	13 ± 6	60	0	63 ± 9	50 ± 9	20 ± 7
15	133	31 ± 8	25 ± 8	19 ± 7	60	33	63 ± 9	44 ± 9	19 ± 7
15	200	31 ± 8	13 ± 6	13 ± 6	60	67	56 ± 9	25 ± 8	9 ± 5
15	267	31 ± 8	9 ± 5	9 ± 5	60	100	28 ± 8	13 ± 6	9 ± 5
22.5	0	84 ± 7	56 ± 9	38 ± 9	60	133	19 ± 7	13 ± 6	9 ± 5
22.5	33	78 ± 7	69 ± 8	32 ± 9	60	200	13 ± 6	3 ± 3	9 ± 5
22.5	67	63 ± 9	56 ± 9	22 ± 7	60	267	9 ± 5	6 ± 5	6 ± 4
22.5	100	50 ± 9	25 ± 8	25 ± 8	−	−	−	−	−

*SE, standard error; means for direct launches were calculated based on different numbers of trials in each. Experiment (Experiment 1 = 2; Experiment 2 = 32; Experiment 3 = 288 trials)*.

### Discussion

Results from Experiment 1 demonstrate that participants use kinematic temporal and spatial information to infer causal relationships in launching events. The absence of Trial × Time or Trial × Space interactions suggests that over the course of the experiment participants did not alter their use of time and space to infer causality. These results provide us with a baseline to examine context effects. If contextual information provided by recent and ongoing exposure to direct causal events plays a significant role in how kinematic temporal and spatial information contribute to causality, increasing participants’ exposure to direct causal events should alter how participants’ use time and space to infer causal relationships in launching events. In contrast, if contextual information provided by increased exposure to direct causal events does not influence our use of time and space to causality, there should be no effect of increased exposure to “causal context.”

## Experiment 2

In Experiment 2, participants were presented with more direct causal launches (25%) than in Experiment 1 (2%). If the contextual experience provided by proportionate exposure to direct causal events modulates participants’ inferences of causality, the temporal, and spatial intervals associated with causal inferences should change with increased presentation of launches depicting direct causal events. Unlike Powesland (1959, increased exposure to causal context was not presented in a series of practice trials before the actual test trials or in a block of example trials between blocks of test trials. Instead, more direct causal events were randomly inserted into the test trials. If Powesland’s (1959) contextual findings generalize to ongoing exposures, participants would use smaller kinematic temporal parameters to infer causal relationship with increased exposure to direct causal launches. Additionally, previous research has yet to demonstrate an influence of contextual information on people’s use of kinematic spatial parameters to infer causal relationships. We predict that space will also be affected by the contextual information of increased exposure to direct causal events. However, if participants are insensitive to the causal context of events, temporal, and spatial parameters associated with causal inferences would not change. Alternatively, if time, but not space, is susceptible to influence from contextual information, increased exposure to direct causal events will only influence participants’ use of kinematic temporal information to infer causal relationships.

In addition to the manipulation of causal context, participants in Experiment 2 were presented with 25% more trials than participants in Experiment 1. Thus, if experience over time plays an important role in the contribution of time and space to causal inferences, participants’ use of this information should change over the course of the experiment and be significantly different between experiments.

### Materials and methods

#### Participants, materials, design, procedures, and analyses

A new group of sixteen participants meeting the same criteria as in Experiment 1 participated in Experiment 2. The design, procedures, and analyses were similar to Experiment 1 except for two modifications. Participants in Experiment 2 viewed the same 98 launches as in Experiment 1, with an additional 30 clearly causal launches. Thus, 25% of 128 trials contained unambiguously causal events. Context condition (i.e., 2 vs. 25% clearly causal events) was included in a separate GLMM, in addition to trial number, angle change, and time delay, to evaluate between group differences in the influence of increased causal context on participants’ use of time and space in inferences of causality. Significant Context × Time and Context × Space interactions would suggest that exposure to different degrees of causal context influenced participants use of time and space when making causal judgments. Significant Trial × Time or Trial × Space interactions would suggest that participants were adjusting their use of time or space through the duration of the experiment when making causal inferences.

### Results

Generalized linear mixed models analysis demonstrated that participants in Experiment 2 used kinematic temporal and spatial information when inferring causality (see Table 1 for parameter estimates, see middle panel of Figure 2). There was no main effect of trial, nor any significant interactions in the model (e.g., Trial × Time, Trial × Space, Time × Space; Table 1). A GLMM evaluating the influence of causal context conditions between Experiment 1 and Experiment 2 demonstrated significant Context × Time and Context × Space interactions (Table 1). All other main effects and interactions in the model were non-significant (Table 1). Negative parameter estimates from significant Context × Time and Context × Space interactions suggest that participants exposed to more direct causal events (25%) were more conservative in accepting time delays and angle deviations in judging causal launching events (see top vs. middle panel of Figure 2; see Table 2 for mean values).

### Discussion

During increased exposure to direct causal events, participants used smaller kinematic temporal *and* spatial parameters to infer causal relationships (i.e., more conservative use of time and space), compared to Experiment 1. These results demonstrate that the use of space, like the use of time, is susceptible to contextual influence in causal inferences. Participants update their use of time and space in judging causality based on recent and ongoing experience with events. The lack of Trial × Time or Trial × Space interactions suggests that this updating was evident across the duration of this experiment in a straightforward manner.

## Experiment 3

Participants in Experiment 3 were presented with more direct causal events (75%) than in the previous two experiments. If participants flexibly use time and space to infer causality and the degree of causal context experienced plays a role in this process, further increasing participants exposure to causal structure should amplify effects of the previous experiment.

### Participants, materials, design, procedures, and analyses

A new group of sixteen participants meeting the same criteria as the previous two experiments participated in Experiment 3. The design, procedures, and analyses were similar to Experiment 1 and 2 with two exceptions. Participants in Experiment 3 viewed a block of trials containing 75% clearly causal launches (*n* = 288) and 25% with varying temporal and spatial parametric combinations (*n* = 96; total trials = 384). Context condition (i.e., 2, 25, 75% direct causal events) was included in a separate GLMM, in addition to trial number, angle change, and time delay, to evaluate between group differences in the influence of increased causal context on participants’ use of time and space to make inferences of causality. Significant Context × Time and Context × Space interactions would suggest that exposure to different degrees of causal context influenced participants use of time and space when making causal judgments. Significant Trial × Time or Trial × Space interactions would suggest that differences between total trial numbers influenced the contribution of time and space to causal inferences.

### Results

**Figure 3:**
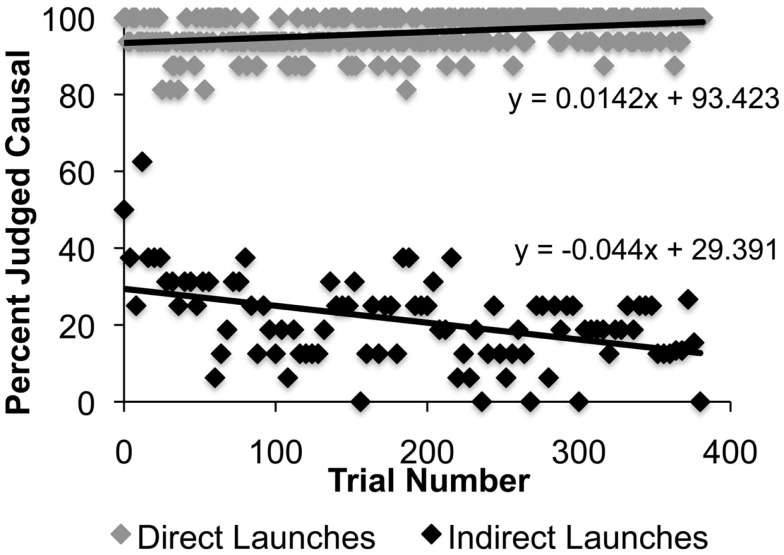
**Percent of trials judged causal as a function of trial number for direct and indirect trial types in Experiment 3**. The positive trend line (slope = 0.014) for direct launches demonstrates that participants were slightly more likely to judge direct launching events as causal over the duration of Experiment 3. In contrast, the negative trend line (slope = −0.04) for indirect launches demonstrates that participants were less likely to judge indirect launching events as causal over the duration of Experiment 3.

Generalized linear mixed models analysis demonstrated that participants in Experiment 3 used kinematic temporal and spatial information to infer causality. There was also a main effect of Trial and both the Trial × Time and Trial × Space interactions were significant. Furthermore, the Time × Space and Trial × Time × Space interactions were significant (see Table 1 for parameter estimates). A main effect of Trial suggests that participants’ judgments of causality changed over the duration of the experiment. The significant negative parameter estimates for the Trial × Time and Trial × Space interactions suggest that as participants were exposed to more trials, they became more likely to reject smaller intervals of time and space as contributing to causality than they were earlier in the experiment (i.e., more conservative). Two distinct trial-based effects are evident when direct and indirect launches are plotted separately (Figure 3). Participants’ consistently judged direct launches as causal throughout the experiment, with a slight increase in the rate of this judgment over time (positive slope in top of Figure 3). In contrast, participants’ judgments of indirect launches became more conservative over time (i.e., more likely to reject smaller intervals of time and space; negative slope in bottom of Figure 3). Thus, effects of trial in Experiment 3 appear to be driving changes in causal judgments on indirect launching events. However, a GLMM analysis containing only indirect launches failed to demonstrate a significant effect of Trial [Parameter Estimate (Est.) = −0.0003, SE = 0.001, *t* = −0.18, *p* = 0.85], Trial × Time (Est. = −2.5E−5, SE = 1.4E−5, *t* = −1.7, *p* = 0.08), Trial × Space (Est. = −3E−5, SE = 5.8E−5, *t* = −0.56, *p* = 0.57), or a Trial × Time × Space interaction (Est. = 5.7E−7, SE = 4.7E−7, *t* = 1.2, *p* = 0.22), making clear interpretation of these data less than straightforward. In terms of the Time × Space interaction, participants in Experiment 3 generally demonstrated a decreased likelihood of causal judgment with increasing time delays. However, for angle deviations between 15° and 45°, participants also demonstrated a slight increase in the likelihood of causal judgment on longer time delay parameters (see Table 2). Thus, certain combinations of temporal and spatial parameters altered participants’ likelihood of making a causal judgment. This pattern likely resulted in the Time × Space interaction in Experiment 3. The positive parameter for the Trial × Time × Space interaction suggests that this pattern was more pervasive as trial number increased in Experiment 3.

**Figure 4:**
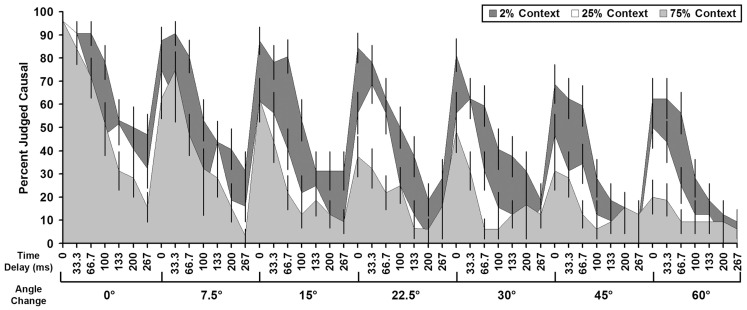
**Difference in the percent of trials judged as causal (*y*-axis) for all combinations of temporal and spatial parameters (*x*-axis) in the 2, 25, and 75% causal context conditions**. Standard error bars are included for each combination of time and space. The smaller area coverage for the 75% context condition (dark gray) demonstrates more conservative use of spatial and temporal parameters vs. the 25% (white) and 2% (light gray) context conditions. Means for direct launches were calculated based on different numbers of trials in each experiment (Experiment 1 = 2; Experiment 2 = 32; Experiment 3 = 288 trials).

A separate GLMM evaluating the influence of causal context conditions between Experiment 1, 2, and 3 demonstrated significant Context × Time and Context × Space interactions (Table 1). There was also a significant Context × Time × Space interaction. All other main effects and interactions in the model were non-significant (see Table 1). Negative parameter estimates from Context × Time and Context × Space interactions suggest that as participants were exposed to more direct causal events, they were more likely to use smaller intervals of time and space to make causal inferences on launching events (Figures 2 and 4; Table 2). Consistent with the graphic depiction of the data in Figure 4, participants exposed to more direct causal launches typically only accepted smaller time delays and spatial angles as causal. The Context × Time × Space interaction was likely driven by the Time × Space interaction in Experiment 3, but not in Experiments 1 or 2. However, this interaction could mean that interactions between time and space were stronger across experiments with more direct causal events (see Table 2; Figure 2). The absence of significant Time × Space interactions in or between Experiments 1 and 2 (Table 1) make the latter hypothesis less likely.

### Discussion

A further increase in exposure to direct causal launches resulted in participants being more conservative in their use of kinematic temporal and spatial information to infer causality (Figures 2 and 4). The significant effects of Trial in Experiment 3, and not the earlier experiments, may have been because of the greater number of trials in this experiment. Figure 3 demonstrates that trial effects were related in part to changes in judgments on indirect launches, with participants less likely to call events “causal” as the experiment proceeded. In contrast, direct launches were more consistently judged as causal than indirect launches. A greater number of trials than were present in Experiments 1 and 2 may have been needed to detect these subtle effects. The trial effect in Experiment 3 is also consistent with the idea that as participants were exposed to more direct launches they judged a lower percentage of other trial types as causal (Figure 4). The Context × Time and Context × Space interactions in the GLMM of the three causal context conditions demonstrates that the context of apprehending events plays a strong role in modulating how kinematic temporal and spatial information contribute to inferences of causality in launching events. These data suggest that participants flexibly update their representation of how kinematic temporal and spatial information relate to causal relationships in events.

## General Discussion

Contextual information plays an important role in how we interpret the relationship between time, space, and causality. The ability to infer causality from kinematic temporal and spatial information is central to our understanding of events in the environment, as well as our ability to predict future outcomes and plan goal-directed actions (Wolff, 2007, 2008). People adjust the temporal and spatial parameters they associate with causality to accommodate the context in which they apprehend launching events (see Table 2; Figures 2 and 4). Others have demonstrated that context influences the contribution of time to inferences of causality (e.g., Powesland, 1959; Buehner and May, 2002, 2003). Our data demonstrate that context effects also extend to the use of spatial information in causal inferences.

Prior work on the effects of foreknowledge (e.g., Schlottmann, 1999; Buehner and May, 2002, 2003) on causal inferences demonstrates that participants flexibly use kinematic information to infer causality. That is to say, people adjust their judgments of the relationship between time and causality based on contextual information, such as how events are framed before they are encountered. Our findings along with those of Gruber et al. (1957) and Powesland (1959) suggest that flexibility in causal inferences also occurs in an ongoing way during the unfolding of events. These effects may arise from top-down knowledge obtained through ongoing observation of changes in the events occurring in our environment. Alternatively, these effects may arise from perceptual anchoring, a form of perceptual adaptation (Helson, 1964). In this view, changes in the use of time to make causal judgments may reflect perceptual adaptive processes responding to prototypical causal or non-causal trials presented during practice. Whereas knowledge-based manipulations are not attributable to perceptual adaptation, experience-based effects could arise from such an effect. As discussed by Hecht (1996), distinguishing between perceptual adaptation and a top-down process for such experience-based effects is exceedingly difficult, if not impossible.

Regardless of the underlying mechanisms, our results suggest that studies using a narrow range of temporal and spatial violations (e.g., the present experiments; Michotte, 1946/1963) produce a rapid decline in the likelihood of making causal judgments for launches with increasing time delays, spatial gaps, or angle deviations. In contrast, studies using a broader range of temporal and spatial violations appear to produce a more gradual decline in the likelihood of endorsing causality in events as violations of time and space increase (e.g., Young et al., 2005). For example, Young et al. (2005) presented participants with launching events with a range of temporal delays between 0 and 2 s, vs. the 0–267 ms range in the present study. Across both studies, a similar pattern of causal judgment was found across the overall range of temporal parameters. That is to say, participants in Young et al. (2005) responded to the 2 s delay much the same as our participants responded to a 267 ms delay. In contrast, a 500 ms delay in Young et al’s. (2005) study was more often judged as causal (∼65%) than our 267 ms delay. Collectively, these results suggest that participants respond to contextual information provided by the overall range of temporal and spatial violations they experience in launching events. The present research also suggests that people are sensitive to contextual information provided by their recent experience with direct causal events. Although the three experiments from the present work contained the same range of temporal and spatial violations, participants were less likely to judge events with longer delays and greater angle violations as causal when they experienced more direct launching events.

Contextually driven changes in people’s use of time and space to infer causality suggest the categories “causal” and “non-causal” are applied flexibly to events. Schwarz (1999) demonstrated that when categories, like “frequent,” “important,” or “successful,” lack clear boundaries, they are malleable and susceptible to contextual influence. Our category of causality might similarly lack clear boundaries and thus lend itself to flexibility in its application to events.

The flexibility of criteria used to make causal inferences may also have implications for its expression in disease. For example, people with paranoid schizophrenia or obsessive-compulsive disorder (OCD) often infer causal relationships where none exists (e.g., Tschacher and Kupper, 2006; Dettore, 2011). In contrast, children with autism can fail to comprehend causal relationships in events in their social environment (e.g., Ray and Schlottmann, 2007). Difficulty integrating contextual information into one’s judgment of causality may play a role in disorders with impaired comprehension of causal relationships in physical and social events. Future studies investigating patients’ ability to flexibly update their representation of the relationship between time, space, and causality in response to changing contextual information will test this hypothesis.

### Conclusion

Our findings show that even in simple mechanical launching events, recent and ongoing contextual information modulates the way that kinematic temporal and spatial information contribute to causal inferences. Situations we encounter in our environment vary considerably. Accounting for contextual information in our representation of causality allows integrations of novel, varied, and relevant information with a person’s own experiences and expectations when making causal inferences. The ability to integrate contextual information into our inferences of causality is likely of adaptive significance.

## Conflict of Interest Statement

The authors declare that the research was conducted in the absence of any commercial or financial relationships that could be construed as a potential conflict of interest.
